# Magnetic Resonance Urogram in Pediatric Urology: a Comprehensive Review of Applications and Advances

**DOI:** 10.1590/S1677-5538.IBJU.2025.0047

**Published:** 2025-02-25

**Authors:** Benjamin Press, Joo Cho, Andrew Kirsch

**Affiliations:** 1 Emory University School of Medicine Department of Pediatric Urology Atlanta GA USA Department of Pediatric Urology, Emory University School of Medicine, Atlanta, GA, USA; 2 Children's Healthcare of Atlanta Department of Pediatric Urology Atlanta GA USA Department of Pediatric Urology, Children's Healthcare of Atlanta, Atlanta, GA, USA; 3 Children's Healthcare of Atlanta Department of Pediatric Radiology Atlanta GA USA Department of Pediatric Radiology, Children's Healthcare of Atlanta, Atlanta, GA, USA

**Keywords:** Magnetic Resonance Imaging, Urography, Pediatrics

## Abstract

Magnetic Resonance Urography (MRU) has emerged as a powerful imaging modality in pediatric urology, offering comprehensive anatomical and functional assessment of the urinary tract without exposure to ionizing radiation. This review provides an in-depth analysis of MRU's technical aspects, clinical applications, advantages, and recent advancements. Traditional imaging techniques, such as ultrasound, voiding cystourethrography, and nuclear scintigraphy, have long been utilized for evaluating pediatric urinary tract anomalies; however, these methods have inherent limitations in anatomical resolution and functional assessment. MRU combines high-resolution anatomical imaging with dynamic functional analysis, making it particularly valuable in evaluating conditions such as hydronephrosis, ureteropelvic junction obstruction, and ectopic ureters. Advancements in MRU technology, including the use of 3T MRI for superior spatial resolution, diffusion-weighted imaging, and dynamic contrast-enhanced imaging, have enhanced its diagnostic capabilities. The ability to assess renal transit times and differential renal function allows for precise evaluation of obstructive uropathies and congenital anomalies. Despite requiring sedation in younger children and longer acquisition times, MRU has demonstrated superior accuracy compared to conventional imaging, reducing the need for multiple diagnostic studies. Recent developments in real-time MRI, faster imaging techniques, and AI-based reconstructions have further optimized MRU's efficiency and diagnostic utility. As MRU continues to evolve, its role in pediatric urology is expected to expand, potentially replacing traditional imaging modalities in select cases. This review highlights the growing significance of MRU in pediatric urinary tract evaluation, emphasizing its potential to improve clinical decision-making and patient outcomes.

## INTRODUCTION

Magnetic Resonance Urography (MRU) is an advanced imaging technique that combines the principles of magnetic resonance imaging (MRI) with specialized protocols to determine anatomy and evaluate function within the urinary tract. MRU provides highly detailed anatomical and superior functional information about the kidneys, ureters, and bladder without the use of ionizing radiation. MRU is particularly valuable in pediatric patients, where minimizing radiation exposure is crucial. The development of MRU began in the early 2000s, with significant advancements over the past two decades (1-12).

Initially, MRU was primarily used in adult patients, but its application in pediatrics has grown as the technology has improved. The refinement of MRU techniques has allowed for better resolution and faster imaging times, making it more feasible for use in children (13, 14). Imaging the urinary tract in pediatric patients is essential for diagnosing and managing various congenital and acquired conditions. Common indications for imaging include neonatal hydronephrosis, ureteropelvic junction obstruction and megaureter, and congenital anomalies such as duplex kidneys and follow-up of vesicoureteral reflux. Traditional imaging modalities like ultrasound (US), voiding cystourethrography (VCUG), and radionuclide scintigraphy have been used extensively, but each has limitations, particularly in providing comprehensive anatomical and functional information. MRU offers several advantages over traditional imaging techniques. It provides high-resolution images that can delineate complex anatomical structures and assess renal function and urinary tract drainage in a single study (15, 16). Unlike computed tomography (CT) scans and nuclear medicine studies (e.g MAG3 and DMSA scans), MRU does not expose patients to ionizing radiation, making it a safer option for repeated imaging in children. MRU combines the diagnostic capabilities of multiple traditional modalities into a single comprehensive exam. In this article, we describe the basics of how magnetic resonance urography is performed in the pediatric population as well as the common indications and relative performance compared to standard imaging modalities in the context of pediatric hydronephrosis.

## TECHNICAL ASPECTS OF MRU

Both 1.5-Tesla (T) and 3T MR scanners may be used in order to perform MRU in pediatric patients. Advantages of 3T MRI include higher signal-to-noise ratio (SNR), which improves the spatial and temporal resolution, critical for detailed anatomical visualization of the urinary tract. This allows for thinner slices and better 3D reconstructions, crucial for evaluating complex anomalies. Higher SNR also improves sensitivity for detecting renal parenchymal and urinary tract abnormalities Furthermore, improved temporal resolution can enhance assessment of renal perfusion and excretion. T2-weighted and other fluid-sensitive sequences benefit also from the higher SNR, which can be advantageous if contrast use is contraindicated. However, artifacts from metal implants, surgical clips, and bowel gas can be worse in a 3T compared to 1.5T, and field signal homogeneity is better at 1.5T, reducing shading artifacts, especially in larger children. Homogeneity of the magnetic field is better at 1.5T, reducing shading artifacts, especially in larger children (17, 18).

A combination of anatomical and functional sequences is used to ensure comprehensive evaluation. Localizer sequences with T2-weighted Half Fourier Single-shot Turbo spin-Echo (HASTE) or SSFSE (Single Shot Fast Spin Echo) provide a reference to guide the placement of imaging planes for the detailed study (19). T2 weighted imaging is a foundation for anatomic imaging, which provides high-contrast images of fluid-filled structures, highlighting the urinary tract. These images allow for visualization of dilated urinary structures (e.g., hydronephrosis), assessment of anatomy in congenital anomalies (e.g., duplex collecting systems, infundibular stenosis), and identification of perirenal fluid collections or cystic lesions. T1 weighted images provided information anatomical structures with a focus on solid organs and tissues, such as the renal parenchyma. Anatomic imaging also includes diffusion weighted imaging (DWI), which detects renal parenchymal abnormalities, such as acute pyelonephritis, identifies restricted diffusion in tumors or abscesses and helps differentiate between obstructive and non-obstructive hydronephrosis. Sequences targeted at the urinary tract include high resolution 2D and 3D T2-weighted images, which when obtained in a 3D fashion allow multiplanar reformatting and can be used to make a variety of reconstructions to aid in anatomic delineations such as ureteral strictures, ectopic ureteral insertions, and fistulas. (e.g., volume rendered and maximum intensity projection images) (14).

Functional MRU is an advanced imaging modality that analyzes functional parameters to determine whether there is physiologically significant obstruction in a dilated collecting system. It utilizes dynamic contrast-enhanced (DCE) imaging by tracking the passage of gadolinium-based contrast agents through the kidneys and into the collecting system. DCE imaging captures various phases, including the arterial phase (renal artery anatomy), the corticomedullary phase (renal parenchyma), and the excretory phase (contrast transit through the ureters and bladder). This approach provides crucial information for quantifying transit times, time to peak, volumetric and Patlak differential renal functions, estimated glomerular filtration rate, and asymmetry index to determine the severity of obstruction.

Quantitative parameters in functional MRU offer a detailed assessment of renal function and excretion, providing invaluable diagnostic information. One key parameter is renal perfusion, which evaluates blood flow through the kidneys using time-intensity curves derived from dynamic contrast-enhanced imaging. This helps identify perfusion deficits caused by conditions like renal artery stenosis or ischemia. Differential renal function (DRF) is another critical measure, determining the functional contribution of each kidney to overall renal output. It is especially useful in cases involving congenital anomalies, such as duplex kidneys, or in compromised kidneys due to various causes of urinary obstruction.

Transit times are parameters that assess the movement of contrast from the glomeruli to the collecting system, providing insights into urinary flow dynamics. Delays in transit often indicate obstructions or impaired renal function. Excretory dynamics, measured during the post-arterial phase of contrast imaging, help visualize and quantify the excretion of contrast material into the ureters and bladder, distinguishing between obstructive and non-obstructive pathologies. There are three measured transit times. Mean transit time (MTT) is the time required for the gadolinium to transit from renal plasma to the tubular system. Calyceal transit time (CTT) is the time required for the contrast to reach the peripheral calyces. Renal transit time (RTT) is the time it takes for the contrast to reach the proximal ureter below the inferior pole of the kidney.

Quantitative assessments, combined with advanced imaging techniques like diffusion-weighted imaging (DWI), provide a comprehensive understanding of both global and regional renal function. Functional MRU allows precise, radiation-free evaluation of the urinary system, making it a powerful tool for diagnosing a wide range of conditions, from congenital abnormalities to post-surgical complications.

MR urography can be performed on either 1.5T or 3T MRI, and the study is divided into three phases. Before the scan, a patient is encouraged to drink clear liquids until approximately 1 hour prior to the scan. Patients are not allowed to eat any solid food starting six hours prior to imaging. Once the patient arrives at the imaging center, an IV access is established, and normal saline bolus is administered at 20 mL/kg over a period of 30 minutes. After adequate hydration, patient is escorted to the MRI and positioned comfortably on the table. Then, sedation is initialed if the patient is nine years or younger. After sedation, Foley catheter is placed in the bladder and Foley bag positioned on the side of the patient, below the bladder for passive drainage. MR sequences are obtained as summarized in Table-1. It is important to note that IV furosemide is injected into the patient approximately 15 minutes prior to dynamic phase imaging to allow maximum pharmacologic effect on the kidneys. To minimize motion artifact during dynamic imaging, the level of sedation is increased several minutes prior to contrast injection. Once the sequences are obtained, data is sent for renal segmentation and analysis at a separate workstation.

**Table 1:** List of MRI sequences and estimated time for performing MR urography.

MRI Sequences		Est Time (min:sec)
Localizer		0:08
HASTE Sagittal FS		0:16
HASTE Coronal FS		0:15
T2 Axial HR FS Kidneys		5:39
**Lasix Given**		
	T1 FLAIR FS Coronal	4:38
	3D T2 Triggered Kidneys/Ureters	5:00
**Increase Sedation**		
	DWI	2:56
	T2 Axial FS Bladder	2:47
**Contrast Injection**		
	3D Dynamic Coronal	10:00
**Decrease Sedation**		
	3D GRE Sagittal	2:50
	3D GRE Coronal	2:11
	PD Axial FS Kidneys	1:55

Advantages of MRU in pediatric hydronephrosis and comparison to other imaging modalities

Ultrasound is the most widely used imaging modality for evaluating the kidneys and bladder both pre- and postnatally. It offers several advantages, including being non-invasive, free of ionizing radiation, real time imaging, portability, cost effective, and typically performed without sedation. US generally provides sufficient detail to assess renal anatomy and parenchymal changes, such as thinning, altered echogenicity, or cysts, making it the primary tool for identifying and grading hydronephrosis. However, US has limitations in visualizing ureters, particularly when they are non-dilated, and is less effective in imaging the mid-ureter and ureterovesical junction. In cases of significant ureteral dilation anatomical distortion and the limited field of view can make it difficult to fully characterize the urinary tract. Additionally, US does not provide functional information about the kidneys, though future techniques using intravascular contrast agents, such as microbubble contrast, may offer insights into differential perfusion without relying on nuclear medicine or MRI-based contrast agents. Factors like bowel gas, body habitus (e.g., scoliosis, obesity), and patient cooperation can also affect image quality.

Diuretic renal scintigraphy studies still considered the "gold standard" worldwide, offer functional insights into the urinary system depending on the radiopharmaceutical used. Diuretic renal scintigraphy with mercaptoacetyltriglycine (MAG3) evaluates differential renal function and drainage, while dimercaptosuccinic acid (DMSA) scintigraphy assesses renal parenchyma and detects scarring. Diethylenetriaminepentaacetic acid (DTPA) provides information on glomerular filtration-based differential renal function and drainage. Although scintigraphy provides limited anatomical detail, it remains the gold standard for functional assessment. These studies expose patients to ionizing radiation but typically do not require sedation.

CT is occasionally used in pediatric urology, mainly for evaluating renal masses and urinary tract stones. Its limited use is due to the associated ionizing radiation exposure. CT urography, commonly performed in adults, is infrequently used in children because it typically requires multiple image acquisitions (e.g., non-contrast, parenchymal/nephrographic, and ureteral/excretory phases). Techniques like dual-energy CT, which generates virtual non-contrast images, or split-bolus CTU, which combines nephrographic and excretory phase information in a single acquisition, can reduce the number of scans. While CT can provide a qualitative assessment of renal function across multiple phases, this approach is often impractical in pediatrics due to the associated radiation dose.

MRU stands out as the optimal imaging modality for pediatric hydronephrosis due to its ability to provide comprehensive anatomical and functional information without exposing children to ionizing radiation. Unlike ultrasound, which is highly operator-dependent and limited in functional assessment, MRU offers consistent, high-resolution images that detail both the structure and function of the urinary tract. Compared to VCUG, which is invasive and primarily focused on the bladder and urethra, MRU is non-invasive and provides a complete overview of the entire upper and lower urinary system, including the kidneys and ureters. Additionally, while nuclear scintigraphy has been traditionally used for functional assessment, it lacks the detailed anatomical resolution that MRU provides and exposes patients to radiation. Furthermore, it is the author's opinion that MRU excels in functional assessment by analyzing series of different transit times and differential renal functions compared to nuclear scintigraphy. This makes it the superior choice for evaluating pediatric hydronephrosis, ensuring accurate diagnosis and effective treatment planning.

## CLINICAL APPLICATIONS

**Figure 1:**
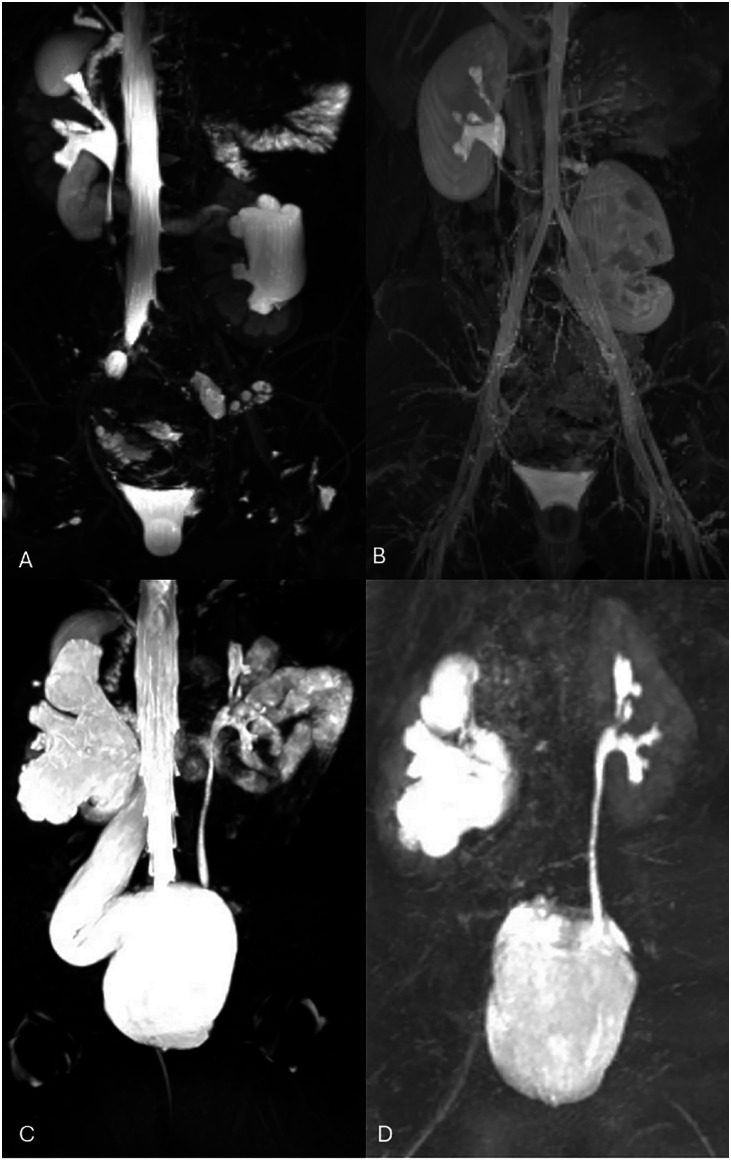
T1 and T2 sequences provided complimentary information in the evaluation of a ectopic left kidney in a 10-month-old girl (A, B) and of an obstructive right megaureter in a 9-month-old girl (C, D).

**Figure 2:**
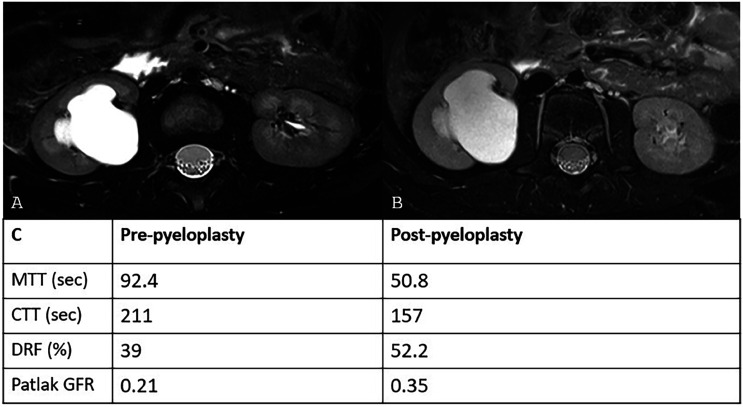
Axial T2-weighted images through the kidneys show similar dilated right renal collecting system in pre- (A) and post-pyeloplasty (B) kidneys. Functional parameters comparing pre- and post-pyeloplasty show normalization of MTT, CTT, differential renal function (DRF), and unit GFR in the right kidney after pyeloplasty (C).

Pediatric urology often involves complex anatomic variants that traditionally require multiple studies such as ultrasound, VCUG, and scintigraphy, for thorough evaluation. MRU has become increasingly popular for its ability to provide a comprehensive assessment of the urinary tract with corresponding functional data for surgical planning and follow up management (Figures 1 and 2).

A) T2 MIP: left ectopic and malrotated kidney with SFU Grade 3 hydronephrosis, normal right kidney. B) T1 Gd-enhanced MIP: Markedly delayed excretion on the left with asymmetric unit pathologic differential renal function (R 65%/L 35%). C) T2 MIP: Right SFU Grade 4 hydronephrosis and hydroureter, normal right kidney. D) T1 Gd-enhanced MIP: Renal transit time is 2 minutes 9 seconds on the left, which is normal. Renal transit time is prolonged on the right, which measures greater than 10 minutes

### Hydronephrosis

The majority of children with antenatal hydronephrosis have non-obstructive etiologies and pelvicaliectasis decreases over time, without intervention (20). More severe cases of prenatal hydronephrosis have higher chances of urinary tract obstruction such as infundibular stenosis, UPJ obstruction, ureteral stricture, or distal ureter obstruction that may require surgical correction. MRU has proved to be a useful tool to distinguish between non-obstructed patulous renal collecting system from actively obstructed upper urinary tract (21). MRU accurately determines the cause of prenatal hydronephrosis and guides management. It has the potential to replace preoperative multi-modality imaging workup by providing detailed renal pathology information that correlates 100% with surgical findings (22, 23). Severe focal UPJ narrowing, renal parenchymal signal hyperintensity, and hyperintense signal around the kidney or renal collecting system on T2-weighted imaging may be indicative of UPJ obstruction. Post-contrast imaging findings decreased peak signal intensity, prolonged time to peak signal, prolonged contrast transit times, and retention of contrast material in the affected kidney correspond to obstructive pattern (24). MRU is particularly useful for evaluating older children experiencing intermittent flank pain and suspected intermittent hydronephrosis or UPJ obstruction caused by a crossing vessel. These children may have normal ultrasound findings when imaging is performed without fluid stress. While renal scintigraphy can assess kidney function dynamically under fluid stress, MRU offers the added benefit of identifying and visualizing the crossing vessel responsible for the obstruction. Studies have shown that MRU is effective in detecting these vessels in pediatric UPJ obstruction, which can be crucial for planning robot-assisted or laparoscopic surgical interventions (25, 26).

### Hydroureter

Ureteric dilatation can be due to a variety of causes in pediatric population, including vesicoureteral reflux, obstructing ureterocele, congenital megaureter, and ectopic insertion. VCUG is the gold standard for diagnosing vesicoureteral reflux, offering excellent visualization of the urethra and reflux grading but involving gonadal exposure to ionizing radiation. Introduced in 1992, magnetic resonance voiding cystourethrography (MRVCU) emerged as a potential alternative with the development of near real-time MR fluoroscopy (27). Although technically feasible, it seems unlikely that MRVCU will gain widespread acceptance in pediatric populations due to the limitations including the difficulty of some patients to void in the supine position and incomplete voiding of some infants and young children secondary to sedation (28). MRU also provides value in evaluation for patients with ureteral stricture of ureterovesical junction (UVJ) obstruction by providing high level of anatomic detail necessary for the diagnosis. MRU has been shown to be the most sensitive for detecting ureteral strictures. In one study, children with mid-ureteral strictures underwent a mean of 2.7 imaging studies with less than half (42%) receiving the correct diagnosis prior to MRI, which lead to a definite diagnosis in all cases (29).

MRU is a highly effective tool for detecting ectopic ureters, offering superior anatomic resolution and the ability to visualize the ureter's course and termination in detail (30-33). This is particularly valuable in cases of complex congenital anomalies. MRU provides both anatomical and functional information without exposing patients to ionizing radiation, making it an ideal choice for pediatric evaluations. Using gadolinium-based contrast agents, MRU enhances visualization of the ureters, allowing for clear identification of abnormal trajectories or ectopic insertions. Multiplanar and 3-dimentional reconstruction imaging enables detailed assessment of pelvic and retroperitoneal structures, helping to distinguish ectopic ureters from other abnormalities. Additionally, MRU can identify coexisting anomalies, such as duplex kidney systems and ureteroceles which are often associated with ectopic ureters. These features make MRU an invaluable diagnostic modality, especially when traditional imaging methods provide inconclusive results.

MRU is not without limitations. Protocols are relatively complex, requiring careful dosing and timing of hydration, furosemide, and gadolinium. MRU scans are also longer than its alternatives, which can take up to an hour to complete. Because of this, in young children or patients who cannot remain still, sedation or general anesthesia may be necessary, adding complexity and risk to the procedure. A Foley catheter is required for the study, which may cause discomfort for the patient. Excessive motion will limit or prevent post-processing of data, and achieving adequate hydration is essential for proper functional data. MRU also has decreased spatial resolution compared to CT urography, making it less effective at detecting small structures or abnormalities. Furthermore, MRU is not as reliable in identifying calcifications and urinary stones, which can be critical in diagnosing certain conditions. Post-imaging processing requires separate software, which is technically challenging to operate. Lastly, most cases can be categorized into decompensated or compensated hydronephrosis with anatomic correlation, but there are occasional cases with parameters that do not align with conventional criteria, which suggest disease processes that has not been fully understood to date. Despite these drawbacks, MRU remains a valuable tool due to its ability to provide detailed anatomical images without the use of ionizing radiation.

## RECENT ADVANCES

Recent advances in pediatric MRU have significantly improved the diagnosis and management of urinary tract disorders in children. Real-time MRI, while primarily used in orthopedic and cardiac imaging, does have applications in observing the dynamic processes in MRU. Real-time MRI uses advanced imaging sequences like radial FLASH MRI and balanced steady-state free precession (bSSFP). These techniques allow for rapid image capture, often in milliseconds, which is crucial for observing processes in motion. The speed of acquisition minimizes motion artifacts and provides clear images of moving structures, such as the heart or joints, without the need for repeated scans (34). Modern real-time MRI employs iterative reconstruction algorithms that enhance image quality and reduce artifacts. This allows for high-resolution images even with rapid acquisition. Iterative reconstruction algorithms process the acquired data in real time, enhancing image quality by reducing noise and correcting for artifacts, resulting in high-resolution images that are crucial for accurate diagnosis and treatment planning, even when the images are captured quickly (35).

Faster imaging techniques in MRI have significantly enhanced the efficiency and quality of scans (36, 37). Parallel imaging, such as SENSE and GRAPPA, utilize multiple receiver coils to simultaneously capture data from different parts of the body, reducing scan times while improving spatial resolution and signal. Compressed sensing leverages the sparsity of image data to reconstruct images from fewer data points, speeding up acquisition and minimizing motion artifacts. Simultaneous Multi-Slice (SMS) imaging captures multiple slices at once, which is particularly beneficial for functional MRI and diffusion MRI, drastically cutting down scan times. Single-shot acquisition techniques, like echo-planar imaging (EPI), acquire the entire image in one rapid scan, reducing the impact of patient movement. Additionally, AI-based reconstructions enhance image quality by predicting and correcting for artifacts and noise, making MRI more efficient. Advanced pulse sequences, such as fast spin-echo (FSE) and turbo spin-echo (TSE), optimize the timing and order of radiofrequency pulses and gradients, further reducing scan times while maintaining high image quality. These advancements collectively make MRI scans faster, more comfortable for patients, and more effective in diagnosing and monitoring urologic conditions.

## CONCLUSIONS

MRU offers one of the most comprehensive assessments of the urinary tract in children, enabling detailed evaluation of the renal parenchyma, collecting systems, ureters, and bladder, while also providing both static and dynamic functional information. This makes MRU a valuable tool for assessing a wide range of pediatric hydronephrosis and congenital urologic abnormalities. Currently, it is generally used as a problem-solving tool when traditional imaging techniques such as US, VCUG, or diuretic renal scintigraphy are not able to provide sufficient information for clinical decision-making. With expanding research and experience MRU will continue to expand its role in evaluating children with genitourinary anomalies.
